# Modeling cancer metabolism on a genome scale

**DOI:** 10.15252/msb.20145307

**Published:** 2015-06-30

**Authors:** Keren Yizhak, Barbara Chaneton, Eyal Gottlieb, Eytan Ruppin

**Affiliations:** 1 The Blavatnik School of Computer Science, Tel Aviv UniversityTel Aviv, Israel; 2 Cancer Research UK, Beatson InstituteGlasgow, UK; 3 The Sackler School of Medicine, Tel Aviv UniversityTel Aviv, Israel; 4 Center for Bioinformatics and Computational Biology, University of MarylandCollege Park, MD, USA

**Keywords:** Cancer metabolism, Metabolic modeling, Genome-scale simulations

## Abstract

Cancer cells have fundamentally altered cellular metabolism that is associated with their tumorigenicity and malignancy. In addition to the widely studied Warburg effect, several new key metabolic alterations in cancer have been established over the last decade, leading to the recognition that altered tumor metabolism is one of the hallmarks of cancer. Deciphering the full scope and functional implications of the dysregulated metabolism in cancer requires both the advancement of a variety of omics measurements and the advancement of computational approaches for the analysis and contextualization of the accumulated data. Encouragingly, while the metabolic network is highly interconnected and complex, it is at the same time probably the best characterized cellular network. Following, this review discusses the challenges that genome-scale modeling of cancer metabolism has been facing. We survey several recent studies demonstrating the first strides that have been done, testifying to the value of this approach in portraying a network-level view of the cancer metabolism and in identifying novel drug targets and biomarkers. Finally, we outline a few new steps that may further advance this field.

## Introduction

Recent cancer genome studies have led to the identification of multiple cancer-associated genes and pathways (Cibulskis *et al*, 2013; Lawrence *et al*, 2014). It is clear now that cancer initiation and progression are controlled by a host of mutational events in these genes, combined together to support cancerous phenotypes. Furthermore, next-generation sequencing technologies have enabled the screening of numerous cancer types and subtypes, uncovering both inter and intratumor heterogeneity (Lawrence *et al*, 2013). Despite this large diversity in dysregulated cellular processes, many key neoplastic events are converged to alter tumor cell metabolism. Indeed, cancer cells were found to have a metabolism that is remarkably different from the tissues from which they originated, due to their high demand for proteins, lipids, nucleotides and energy, all necessary for enhanced growth and proliferation (Vander Heiden *et al*, 2009). This fundamental characteristic of cancer cells has led to the development of the first chemotherapy treatment, methotrexate, already in the early 1950s (Li *et al*, 1956), in an attempt to target cancer cell proliferation. This drug is designed as an antimetabolite that interferes with the use of folic acid by cancer cells, thus blocking DNA synthesis and halting cell growth. This common denominator among cancer cells together with additional accumulating evidences reviewed below has recently led to the recognition of altered tumor metabolism as one of the hallmarks of cancer (Hanahan & Weinberg, 2011).

Cellular metabolism is finely tuned by integrating signals from the intracellular and extracellular environments. The metabolic switch promoting deregulated growth is often triggered by mutations in signaling pathways that rest at the crux of anabolic and energetic homeostasis, such as HIF-1α, PI3K/AKT, mTOR and AMPK (Shaw & Cantley, 2006; Guertin & Sabatini, 2007; Wise *et al*, 2008; Semenza, 2010). The mutated pathways result in constitutively active growth signals that induce cells to proliferate uncontrollably. In addition to the intracellular genetic modifications, the abnormal environmental conditions also play a major role in modifying cellular metabolism. Heterogeneity in oxygenation, PH levels and nutrient availability are combined with intrinsically altered tumor metabolism, optimizing for a continuous supply of building blocks and redox potential that allow cancer cells to survive and proliferate under strict selective pressure (Cairns *et al*, 2011).

Recent years have significantly advanced our understanding of the genetic and molecular events underlying the metabolic functional phenotype of cancer cells. This has been achieved due to the considerable leap forward in omics measurement technologies, enabling the genome-wide characterization of different altered cellular processes. Accumulating data of gene sequences and gene methylation patterns, gene, protein and microRNA expression measurements, as well as metabolites levels, have revealed a comprehensive and complex picture of dysregulated cellular processes. Nonetheless, the entire metabolic network is comprised of more than a hundred different subsystems, spanning a few thousands of biochemical transformations. To comprehensively understand how the different cellular components interact with each other, as well as figuring how the metabolic network responds to different genetic and environmental perturbations as a whole, computational tools come in hand. In particular, computer simulations enabling the investigation of the network's state under diverse conditions and on a genome-wide level are helpful for studying both normal and cancerous cellular metabolism, and for advancing our ability to identify potential drug targets and biomarkers. Following, this review will discuss our current knowledge of altered tumor metabolism and the challenges in modeling these alterations, through the integration of high-throughput molecular data with state-of-the-art metabolic modeling approaches.

## Metabolic alterations associated with cancer

**Figure 1:**
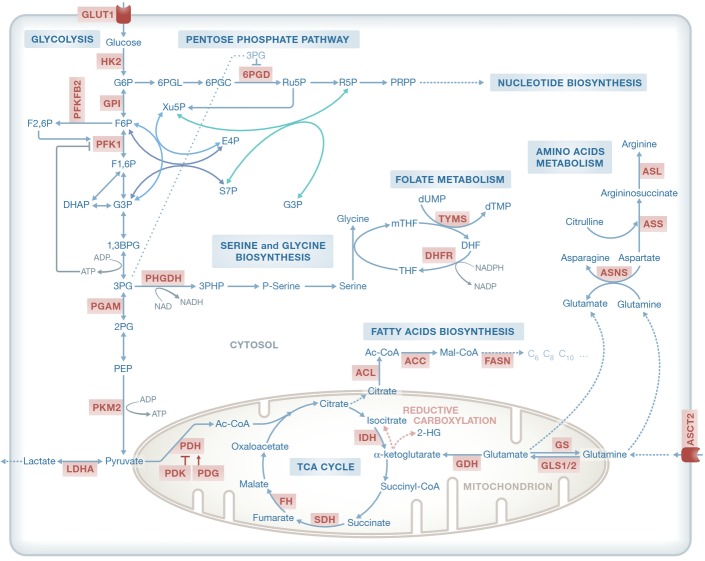
Central metabolic pathways and their association with key metabolic enzymes Enzymes marked in red have been implicated with tumor initiation and progression and/or serve as potential therapeutic targets. G6P, glucose-6-phosphate; F6P, fructose-6-phosphate; F1,6P, fructose-1,6-bisphosphate; F2,6P, fructose-2,6-bisphosphate; G3P, glyceraldehyde 3-phosphate; 1,3BPG, 1,3 biphosphoglycerate; 3PG, 3-phosphoglycerate; 2PG, 2-phosphoglycerate; PEP, phosphoenolpyruvate; 3PHP, 3-phosphohydroxypyruvate; Ac-CoA, acetyl-CoA; 6PGL, 6-phospho-glucono-1,5-lactone; 6PGC, 6-phospho-D-gluconate; Ru5P, ribulose 5-phosphate; R5P, ribose 5-phosphate. PRPP, 5-phospho-alpha-D-ribose 1-diphosphate. S7P, sedoheptulose 7-phosphate; Xu5P, xylulose 5-phosphate; E4P, erythrose 4-phosphate; THF, tetrahydrofolate; mTHF, 5,10-methylenetetrahydrofolate; DHF, dihydrofolate; Mal-CoA, malonyl-CoA; αKG, α-ketoglutarate; dTMP, deoxythymidine monophosphate; dUMP, deoxyuridine monophosphate; TCA, tricarboxylic acid; GLUT1, glucose transporter 1; HK2, hexokinase 2; GPI, glucose-6-phosphate isomerase; PFKFB2, 6-phosphofructo-2-kinase; PFK1, phosphofructokinase 1; PGAM, phosphoglycerate mutase; PKM2, pyruvate kinase M2 isoform; LDHA, lactate dehydrogenase A; PHGDH, phosphoglycerate dehydrogenase; PDH, pyruvate dehydrogenase; PDK, pyruvate dehydrogenase kinase; FH, fumarate hydratase; SDH, succinate dehydrogenase; IDH, isocitrate dehydrogenase; GDH, glutamate dehydrogenase; GLS, glutaminase; GS, glutathione synthetase; ASCT2, solute carrier family 1, member 5; ACL, ATP citrate lyase; ACC, acetyl-CoA carboxylase; FASN, fatty acid synthase; ASNS, asparagine synthetase; ASL, argininosuccinate lyase; ASS, argininosuccinate synthetase; DHFR, dihydrofolate reductase; TYMS, thymidylate synthase.

To set up the stage for our discussion, we first provide a brief overview of the metabolic alterations reported to occur in cancer. For more detailed reviews of the latter see, Cairns *et al* (2011), Vander Heiden *et al* (2009) and Ward and Thompson (2012). One of the most conspicuous features of cancer metabolism was already discovered more than fifty years ago by Otto Warburg, showing that most cancer cells utilize high amounts of glucose and secrete it as lactate even in the presence of oxygen, a phenomenon that is referred to as aerobic glycolysis or “the Warburg effect” (Warburg, 1956). This is in difference from normal cells that metabolize glucose in the mitochondria via the tricarboxylic acid (TCA) cycle and revert to anaerobic metabolism only under low oxygen conditions. Today, this dramatic increase in glucose uptake by cancer cells is exploited clinically to visualize tumors by (^18^F)-2-deoxy-D-glucose positron emission tomography (FDG-PET) (Som *et al*, 1980). Following these early discoveries, the role of glycolysis in cancer cells has been studied extensively and several glycolytic reactions were found to be key regulators of cancer metabolism (Fig1).

Beyond the Warburg effect, major alterations in cancer have been identified in key pathways involved in the production of key biomass components. As expected given the highly proliferative nature of cancer cells (and evidenced by antimetabolite-based chemotherapy treatment), the biosynthesis of building blocks for nucleotide synthesis, as well as NADPH by the oxidative pentose phosphate pathway (PPP, branching from glycolysis), is essential in rapidly proliferating cells. Another pathway that branches from glycolysis is serine biosynthesis, which is crucial for amino acids, lipids and nucleotide synthesis. The up-regulation of this pathway has been associated with the ability of breast cancer cells to metastasize (Pollari *et al*, 2011). Furthermore, a functional genomics screen found that some breast cancer cells rely on endogenous serine production to sustain proliferation. Importantly, the gene encoding for phosphoglycerate dehydrogenase (PHGDH), the enzyme that catalyses the first committed step of serine biosynthesis, is amplified and highly expressed in some cancers, and melanoma and breast cancer cells with PHGDH amplification divert large amounts of glucose-derived carbons into serine and glycine biosynthesis (Locasale *et al*, 2011; Possemato *et al*, 2011) (Fig1).

Many cancer cells undergoing aerobic glycolysis require glutamine carbons to replenish the TCA cycle and sustain accelerated anabolism. In addition, glutamine is also an important nitrogen source for cells (DeBerardinis *et al*, 2008). Glutamine can be deaminated by one of the two glutaminases (GLS1 or GLS2) producing glutamate and ammonia. Under some conditions (e.g. hypoxia), α-ketoglutarate produced from glutamate can undergo reductive carboxylation to generate citrate, oxaloacetate and acetyl-CoA to support anabolic processes anaerobically (Fig1) (Mullen *et al*, 2012). As expected, GLS was found to be overexpressed in a number of tumors, and its inhibition delays tumor growth (Lobo *et al*, 2000; Wise *et al*, 2008).

The role of metabolism in cancer is not limited to the metabolic adaptation to environmental changes or higher proliferation rates. In fact, mutations affecting key metabolic pathways have recently been found in hereditary forms of cancer or shown to increase tumor predisposition, revealing that aberrant metabolism could also be, in some cases, the cause of cancer. Thus, mutations in any of the genes encoding succinate dehydrogenase (SDH) complex subunits were found to be the underlying cause of hereditary paraganglioma, a neuronal crest-derived cancer syndrome (Frezza *et al*, 2011a). Soon after this seminal discovery, fumarate hydratase (FH), the enzyme that converts fumarate to malate, was found mutated in hereditary leiomyomatosis and renal cell cancer (HLRCC) (Kiuru *et al*, 2002; Tomlinson *et al*, 2002). Mutations in these TCA cycle enzymes force cells to rely on a truncated TCA cycle and to accumulate high amounts of succinate or fumarate. It is thought that the resulting stabilization of the oxygen-dependent labile subunit of the hypoxia-inducible transcription factor HIFα, even in the presence of oxygen, gives rise to a pseudo-hypoxic and aerobic glycolysis phenotypes. Another key TCA cycle-related enzyme that was found to be mutated in cancer cells is isocitrate dehydrogenase (IDH) (Fig1). An integrated genomic analysis found recurrent heterozygous mutations in the active site of IDH1 and IDH2 isoforms in high proportion of low-grade glioma and acute myeloid leukemia (AML) patients (Parsons *et al*, 2008; Mardis *et al*, 2009; Yen *et al*, 2010). It was shown that mutant IDH not only has reduced capacity to convert isocitrate to α-ketoglutarate but also acquires a novel reductive activity utilizing α-ketoglutarate to produce 2 hydroxyglutarate (2HG) (Dang *et al*, 2009), which is tumorigenic in glioma and AML. Specific chemical inhibitors against mutant IDH1 and IDH2 have been designed and are currently tested in clinical trials (Wang *et al*, 2013). Taking these findings together, fumarate, succinate and 2HG have been dubbed as “oncometabolites,” giving rise to the possibility that other oncometabolites exist and await discovery.

### Targeting tumor metabolism

As identifying new cancer drug targets is one of the main goals of metabolic modeling in cancer, let us review the current state of efforts to target cancer metabolism in the clinic in some detail. The great number of dysregulated metabolic pathways provides the opportunity for targeting these pathways pharmacologically. A major challenge is however that the vast majority of metabolic pathways used by cancer cells are also essential for the survival of normal ones, as reflected by the undesirable side effects of several chemotherapy agents. Nonetheless, the presence of tumor-specific enzyme isoforms or changes in the activity of a pathway may allow preferential targeting of cancer cells. Indeed, the therapeutic effects of targeting several metabolic enzymes have been investigated in recent years. For instance, glycolytic inhibitors such as GLUT1 inhibitor and 2-deoxyglucose underwent clinical trials (Chan *et al*, 2011; Cheong *et al*, 2012; Gautier *et al*, 2013). Their effect though was found to be limited, potentially due to the strong increase in glutaminolysis displayed by some tumors, and the ability of tumors with functional mitochondria to produce ATP by oxidative phosphorylation. Several inhibitors of amino acid metabolism have also been studied. The main targeted amino acid is glutamine, which can be depleted directly from the blood of cancer patients. Phenylacetate reduces glutamine availability thus inhibiting cancer cell proliferation and promoting differentiation (Samid *et al*, 1993; Wise & Thompson, 2010). However, the removal of glutamine directly from the plasma may also increase the rate at which the body depletes its own muscle stores (cachexia). Another approach is to target GLS directly (Seltzer *et al*, 2010). Further to glutamine, asparagine and arginine biosynthesis can also be targeted by different compounds. Although asparagine is not usually an essential amino acid in humans due to the presence of asparagine synthetase (ASSN), certain tumor types like leukemia have little ASSN activity and require exogenous asparagine (Fig1). This has led to the use of asparaginase, the enzyme that converts asparagine to aspartate and ammonia, for the treatment of childhood acute lymphoblastic leukemia (ALL) (Haskell *et al*, 1969; Pieters *et al*, 2011). Likewise, while in normal tissue arginine is not an essential amino acid, some hepatocellular carcinoma (HCC), mesothelioma and melanomas do not express argininosuccinate synthetase (ASS) and therefore are auxotrophic for arginine and sensitive to its depletion in plasma (Fig1). Arginine deiminase has proved beneficial in the treatment of unresectable melanoma, and it is currently being tested in several other tumor types (Feun & Savaraj, 2006; Delage *et al*, 2010).

Going beyond amino acid metabolism, several inhibitors of fatty acid synthesis have also been developed and studied. Endogenous fatty acids are synthesized from TCA cycle-derived citrate and NADPH, which can be produced by the PPP and other enzymes. Once in the cytosol, citrate is broken down into acetyl-CoA and oxaloacetate by ATP citrate lyase (ACL). Fatty acid synthesis starts with acetyl-CoA carboxylase (ACC) converting acetyl-CoA to malonyl-CoA, and this is followed by a series of steps in which malonyl-CoA and acetyl-CoA are converted to palmitate by fatty acid synthase (FASN) (Fig1). Many tumors therefore express high levels of FASN, including breast, colorectal and endometrial cancers (Alo *et al*, 1996), and FASN inhibitors either kill tumor cells directly or sensitize them to other therapies such as 5-fluorouracil and trastuzumab (Herceptin) (Kridel *et al*, 2004; Menendez *et al*, 2006; Vazquez-Martin *et al*, 2007). The inhibition of other enzymes in the *de novo* lipogenic pathway, such as ACL, choline kinase, ACC, monoglyceride lipase (MGLL) and 3-hydroxy-3-methylglutaryl-CoA reductase (HMGCR), has proved effective as cancer treatment in preclinical settings and these enzymes are in the focus of drug development, and some of them, for example, statins, are currently undergoing clinical trials (Brusselmans *et al*, 2005; Glunde *et al*, 2005; Hatzivassiliou *et al*, 2005; Nomura *et al*, 2010; Bjarnadottir *et al*, 2013).

### Mapping the cancer metabolome

One of the most prominent technology advancements for studying dysregulated tumor metabolism has been the development of metabolomics, a discipline that aims to measure the concentration and relative abundance of small molecule metabolites (< 1.5 kDa) present in biological systems (e.g. cells, tissues or body fluids) and is currently allowing for the simultaneous measurement of hundreds of metabolites (Dunn *et al*, 2005; Lane *et al*, 2009). The use of metabolic profiling in cancer provides an additional layer of patho-physiological information beyond genomic data. Initial metabolomics approaches were based on nuclear magnetic resonance (NMR) but they are now complemented with the use of mass spectrometry (MS), which provides higher sensitivity and a wider range of metabolites detection (Griffiths *et al*, 2010). MS also offers the possibility to perform targeted analyses of metabolic pathways by using ^13^C-labeled metabolites such as glucose and glutamine. This strategy allows for the measurement of intracellular metabolic fluxes and, by making use of partially labeled substrates, for the identification of alternative metabolic pathways (Zamboni & Sauer, 2009). By applying these recent advances in the context of cancer research, metabolic alterations have been observed in a wide variety of tumors, identifying adaptations and vulnerabilities that open new possibilities for the development of cancer therapies.

For instance, LC-MS has been used to study the metabolic alterations associated with the M2 isoform of pyruvate kinase, showing significant differences in glycolytic intermediates (Christofk *et al*, 2008). The same technology was used later on to show that these glycolytic metabolites are fed into serine synthesis, allowing them to proliferate in serine-depleted medium (Ye *et al*, 2012). Other metabolomics flux experiments have employed GC–MS to trace central carbon metabolism. Such studies include the finding that the reductive metabolism of α-ketoglutarate contributes to *de novo* lipogenesis (Metallo *et al*, 2012), the characterization of FH-deficient cells in renal cancer (Frezza *et al*, 2011b), the study of glutamine dynamics in pancreatic ductal adenocarcinoma (PDAC) (Son *et al*, 2013), as well as the study of glutamine-associated changes in glioma cells during impaired mitochondrial pyruvate transport (Yang *et al*, 2014). Metabolomic approaches have been additionally used to detect cancer-specific biomarkers in body fluids. This includes the discovery of long-chain fatty acids in the serum of colorectal cancer patients (Ritchie *et al*, 2010); significant changes in amino acids, bile acids and polar lipids in plasma samples of pancreatic cancer patients (Urayama *et al*, 2010); increased levels of sarcosine in urine samples of prostate cancer patients (Soliman *et al*, 2012); and more (Armitage & Barbas, 2014).

Clearly, a great amount of data describing the metabolic alterations in cancer cells has gathered in recent years, and there is a growing need for its analysis and contextualization on a genome-wide cellular level. A central key approach for addressing these challenges is genome-scale metabolic modeling (GSMM), as reviewed below.

## Genome-scale modeling of cellular metabolism

One of the ultimate goals of Computational Systems Biology is to build an *in silico* model of a living cell that will include all its components and will have a predictive value in simulating all cellular processes. A key difficulty is the lack of sufficient comprehensive knowledge on the pertaining biological processes and associated detailed kinetics. However, despite these difficulties, there is one domain where under simplifying assumptions, and due to two hundred years of biochemistry research, we are able to make first meaningful steps toward realizing this *in silico* vision, and that is cellular metabolism (Kuepfer, 2010). Metabolism is by now the most studied and well-known cellular process across many species, including humans. Over the last decade, recent strides in the computational study of metabolism have enabled its computational investigation on a genome scale in an accelerating pace (Herrgard *et al*, 2008; Bordbar & Palsson, 2012; Mardinoglu & Nielsen, 2012; de Oliveira Dal'Molin & Nielsen, 2013; Bordbar *et al*, 2014).

As reviewed above, recent technological advancements have enabled the genome-wide quantification of gene, enzyme and metabolite levels, thus providing cues to an organism's metabolic state. However, despite this considerable progress, the most direct measure of activity in a metabolic network, the reaction flux rates, can be measured today for only a few dozens of reactions in central metabolism (Niklas *et al*, 2010). The analysis of GSMMs aims to bridge this gap and facilitate the prediction of the network's inner and outer (uptake and secretion) flux rates, thus characterizing the organism's metabolic state on a large scale. Furthermore, GSMM enables the integration of various omics data to obtain context-specific metabolic descriptions, and the simulation of different genetic and environmental perturbations under which the metabolic state can be iteratively re-evaluated.

Genome-scale metabolic model reconstruction is a manual, bottom-up process, in which all the biochemical transformations taking place within a specific target organism or cell are assembled into a metabolic network (Thiele & Palsson, 2010). This network is represented mathematically by a stoichiometric matrix that comprises the stoichiometric coefficients of the metabolic reactions included in the network, and is concomitantly accompanied by a detailed mapping of the genes and proteins to their catalyzed reactions (Orth *et al*, 2010). GSMMs typically form complex models encompassing thousands of genes, proteins, reactions and metabolites.

The analysis of GSMMs is performed via a constraint-based modeling (CBM) approach that imposes a set of physico-chemical constraints on the space of possible metabolic behaviors, including mass balance, thermodynamic (directionality) and maximal flux capacity constraints, while optimizing for a cellular objective function such as maximization of biomass yield or ATP production. The latter is conventionally done via a flux balance analysis (FBA) method. This approach has been extensively and quite successfully applied for more than a decade now to study the metabolism of microorganisms and has been rapidly expanding to dozens of manually curated models for both pro- and eukaryotes (Monk *et al*, 2014).

Despite its considerable predictive signal, it should be acknowledged that the CBM approach makes a few simplifying assumptions to achieve modeling on a genome scale. First and foremost, it assumes that the system modeled is in a quasi-steady state; that is, while internal metabolites may be generated and consumed, their overall levels remain unchanged (while metabolites that are exchanged with the environment may be taken up or secreted). This assumption needs to be made since the kinetic parameters governing the dynamics of the thousands of enzymes in the network are mostly unknown. Second, to obtain a physiological meaningful flux space, an additional objective function needs to be assumed. By and large two different classes of objective functions are assumed—(a) maximizing an assumed “cellular” objective or (b) maximizing the fit between the predicted metabolic state and context-specific molecular omics data. As for (a), maximizing biomass production (a corollary of proliferation rate) is typically used and is appealing in the context of modeling proliferating cells like bacteria and cancer cells. Regarding (b), a variety of approaches exist aiming to best fit the predicted metabolic state to measured flux data, transcriptomics and proteomics, or a combination of the latter (Machado & Herrgård, 2014). A detailed discussion of the latter is beyond the scope of this review, but see some related notes in brief in Box 1. Furthermore, it should be explicitly noted that the models built encompass just the enzymatic reactions that directly modify the metabolites and thus, at least in the context of human metabolism (and in most bacterial models), do not explicitly include interconnected cellular processes such a transcriptional regulation and signaling pathways that regulate metabolism. Including the latter information raises serious computational challenges as assuming steady state is problematic in this context, but even more so, they are simply yet not known at a sufficient level of details. Additionally, while when simulating cell line experiments the growth media is well characterized, regrettably, in simulating *in vivo* systems (like the metabolism of healthy or tumor tissue) the environment is not well characterized and one needs to make some bold assumptions regarding its composition. Finally, in the key application of GSMMs to predict new cancer drug targets, one should note that many relevant factors are actually out of the scope of such an endeavor, including the “druggability” of a predicted target, its cellular localization, its three-dimensional structure and its potential binding with known classes of inhibitors (Hopkins & Groom, 2002; Bunnage, 2011).

**Box 1: Building tissue/cell-specific human GSMMs**

**Figure 2:**
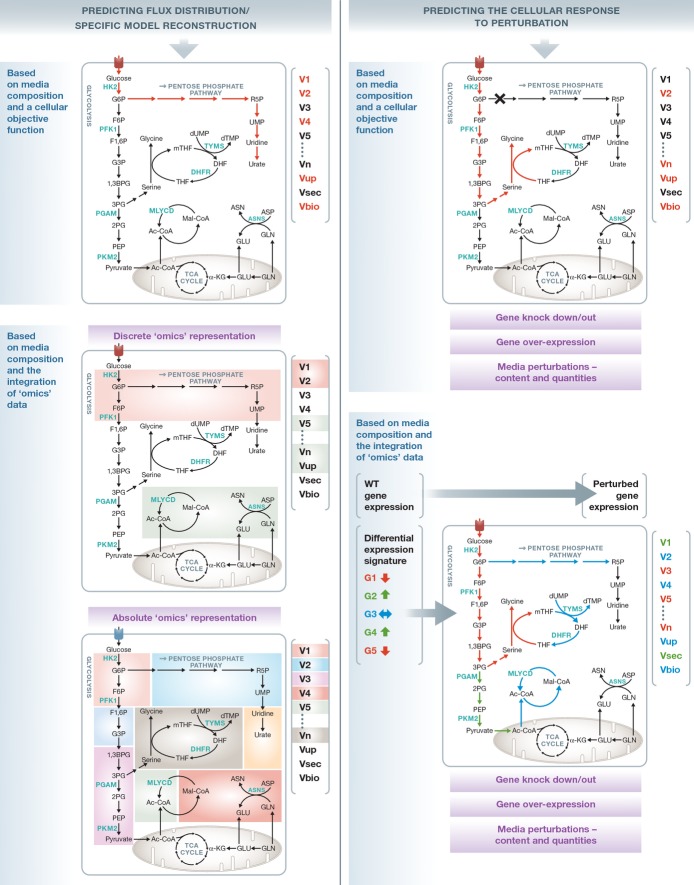
Genome-scale metabolic modeling as a platform for predicting flux distributions and simulating cellular perturbations Genome-scale metabolic modelings (GSMMs) provide an opportunity to characterize a cellular metabolic state by predicting the distribution of the network's reaction flux rates on a genome-scale level. For the analysis of microorganisms, this has been mostly achieved by assuming a pre-defined cellular objective function such as maximization of biomass yield or ATP production (left section, upper panel). Such an objective function cannot always be assumed when analyzing human metabolism, and therefore, omics data are utilized to derive a reduced specific model or characterize a metabolic flux state that best fits the context-specific omics data. The data can be used either in a discrete manner (left section, middle panel), trying to activate the flux thorough reactions associated with highly expressed genes (green) while removing those associated with lowly expressed genes (red), or constraining the model more quantitatively by considering the absolute expression levels (as depicted by the different colors, left section, lower panel). The network can be further studied by simulating genetic and environmental perturbations (right section). Similarly, the flux through the perturbed network can be derived based on a pre-defined objective function (right section, upper panel) or by utilizing the omics data to define the differential expression signature that can then be used to constrain the model in various ways (right section, lower panel).

In general, methods for integrating omics datasets can be classified into those that use a discrete representation of the input data and those that utilize a more quantitative approach:

The first type categorizes the model's reactions into two groups: those associated with highly and those associated with lowly expressed genes. They then apply different types of objective functions aiming to maximize the similarity between this discrete representation and the model's reaction activity state (Becker & Palsson, 2008; Jerby *et al*, 2010; Shlomi *et al*, 2011; Agren *et al*, 2012; Wang *et al*, 2012b) (Fig2). This discrete representation of the expression state might not be sensitive enough for modeling the differences between cells that exhibit only subtle variations in their expression level. Despite some limitations, these approaches have been successfully used as a basis for generating context-specific models of tissues and cells through which both normal and diseased human metabolism have been studied (Bordbar & Palsson, 2012; Mardinoglu *et al*, 2013b; Oberhardt *et al*, 2013).

The second, non-discretized approach utilizes the absolute gene expression levels to derive a flux description of a specific metabolic state (Lee *et al*, 2012), or for constraining reactions' maximal flux capacity for the purpose of building a specific model (Colijn *et al*, 2009; Fig2). While these approaches maintain the basic structure of the network and are more sensitive to subtle differences in expression levels, their drawback is in their underlying implicit assumption that there is a strong monotonic positive association between gene expression levels and flux rates, an assumption that is known to hold only partially (Bordel *et al*, 2010). Applying this approach while utilizing proteomic data can potentially improve model accuracy. These approaches have so far mostly been applied for studying microorganisms. Their application to the study of higher organisms in the context of mammalian physiology and cancer metabolism has only recently been established (Yizhak *et al*, 2014a).

### Genome-scale modeling of human metabolism

Genome-scale metabolic modelings of human metabolism (Table1) have been reconstructed to represent the collection of all the metabolic reactions known to occur in human cells (Duarte *et al*, 2007; Ma *et al*, 2007; Mardinoglu *et al*, 2013a, 2014; Thiele *et al*, 2013). These models have been utilized for modeling both normal and diseased human metabolism, as comprehensively reviewed by Bordbar and Palsson (2012), Mardinoglu and Nielsen (2012). In contrast to the modeling of microorganisms, two crucial points should be taken into consideration when utilizing these human reconstructions: (i) First, the models are not specific to any tissue or cell type. As they encompass the set of all possibly occurring human metabolic reactions, their solution space contains multiple feasible metabolic behaviors that should be further constrained to achieve a level of cell or tissue specificity; (ii) second, the objective function(s) of different human tissues and cells is more difficult to determine (or perhaps even does not exist), especially for those cells that are non-proliferating (and hence maximal biomass yield cannot be assumed). Considering these challenges, the question is then how can we utilize these reconstructions to study normal and diseased human metabolism?

**Table 1:** Human model reconstructions and their usage in cancer metabolism. The table describes the size of the different reconstructions and their specific application in the study of different cancer cells and tissues.

Human model reconstruction	Size			Cancer type	Application	References
	Genes	Reactions	Metabolites			
Recon 1 (Duarte *et al*, 2007)	1,905	3,742	2,766	Generic	Studying the association between cell proliferation and the Warburg effect	Shlomi *et al* (2011)
				Generic	Pathway contribution to NADPH production in cancer	Fan *et al* (2014)
				Generic	Identification of cancer-selective drug targets	Folger *et al* (2011)
				Generic	Predicting combinations of anti-cancer drugs with minimal side effects	Facchetti *et al* (2012)
				26 tumor tissues	Identifying cancer-specific metabolic pathways	Wang *et al* (2012b)
				Liver cancer cell line	Identifying P53-associated metabolic changes	Goldstein *et al* (2013)
				The NCI-60 cell line collection	Studying the association between cell proliferation and nutrients uptake rates	Dolfi *et al* (2013)
				Breast cancer	Studying the metabolic differences associated with tumor stage and type	Jerby *et al* (2012)
				Clear cell renal cell carcinoma (ccRCC)	Identifying synthetic lethal interaction in FH-deficient cells	Frezza *et al* (2011b)
				The NCI-60 cell line collection	Predicting drug-reaction interactions	Li *et al* (2010)
				The NCI-60 cell line collection and breast/lung cancer clinical samples	Personalized prediction of metabolic phenotypes and identification of selective drug targets	Yizhak *et al* (2014a)
				The NCI-60 cell line collection	Association of the Warburg effect with cell migration and identification of anti-migratory drug targets	Yizhak *et al* (2014b)
				Hepatocellular carcinoma	miRNA was simulated to predict their ability to reduce cancer cell growth	Wu & Chan (2014)
The Edinburgh Model (Ma *et al*, 2007)	2,322	2,823	2,671	Colon and breast cancer cell lines	Metabolomic network correlations	Kotze *et al* (2013)
Recon 2 (Thiele *et al*, 2013)	2,194	7,440	5,063	Nine cancer types (TCGA/CCLE)	Identification of oncometabolites	(Nam *et al*, 2014)
HMR (Mardinoglu *et al*, 2013a, 2014)	3,668	8,181	9,311	16 cancer tissues	Identifying cancer-specific metabolic features	(Agren *et al*, 2012)
				Breast, bladder, liver, lung and renal cancer	Topological analysis of ccRCC-specific metabolic processes	Gatto *et al* (2014)
				Hepatocellular carcinoma	Personalized model reconstruction and selective drug target identification	Agren *et al* (2014)
				15 cancer cell types	Studying the topological features of anti-cancer metabolic drugs	Asgari *et al* (2013)

### Simulating genetic and environmental perturbations

Once a specific metabolic model has been reconstructed, it can be utilized to predict cellular responses to genetic and environmental perturbations. The set of genetic perturbations that can be simulated via a GSMM includes both complete (knockout) and partial (knockdown) gene deletions (Orth *et al*, 2010), as well as gene over expression (Wagner *et al*, 2013). Environmental perturbations may be simulated by changing media composition, modifying the quantities of available metabolites as well as enforcing their uptake into the cell (Mo *et al*, 2009) (Fig2). Another type of perturbation is at the *intracellular* metabolite level, where a metabolite deficiency is simulated by its removal from the network (Kim *et al*, 2007). The various perturbations described above can be simulated in all possible combinations, and each time the resulting metabolic state of the cell can be re-evaluated. However, the question is what can we assume about the cellular objective function following such perturbations?

Similar to the simulation of wild-type states, the maximization of biomass yield and ATP production have been extensively used for evaluating the post-perturbation metabolic state (Orth *et al*, 2010), both in microorganism and in cancer cells (Fig2). However, alternative objective functions have also been applied, suggesting that in the perturbed state the cell tries to minimize the deviation from its previous wild-type state (Segre *et al*, 2002; Shlomi *et al*, 2005). Interestingly, it was shown that while the first approach represents the outcome of long-term evolutionary pressure, the second one is more suitable for cases that do not possess a mechanism for immediate regulation of fluxes toward the optimal growth configuration (Segre *et al*, 2002). Despite the fact that these approaches do not consider any condition-specific high-throughput data, they have been successfully used for various drug discovery applications (Kim *et al*, 2010; Shen *et al*, 2010; Folger *et al*, 2011), as well as metabolic engineering tasks (Bro *et al*, 2006; Anesiadis *et al*, 2008), reductive evolution simulations (Pál *et al*, 2006; Yizhak *et al*, 2011), gene essentiality predictions (Duarte *et al*, 2004; Oh *et al*, 2007; Orth *et al*, 2011) and more (Oberhardt *et al*, 2009). Nonetheless, the era of large-scale omics data provides an opportunity for determining the perturbed state without the need to assume a pre-defined objective function (Fig2). Yizhak *et al* (2013) have developed a new algorithm that utilizes source and target gene expression data to predict perturbations that are most likely to transform the metabolic state from one state to the other. The algorithm was applied to study yeast and mammalian aging and led to the identification of novel lifespan-extending genes.

## Genome-scale modeling studies of cancer metabolism

**Figure 3:**
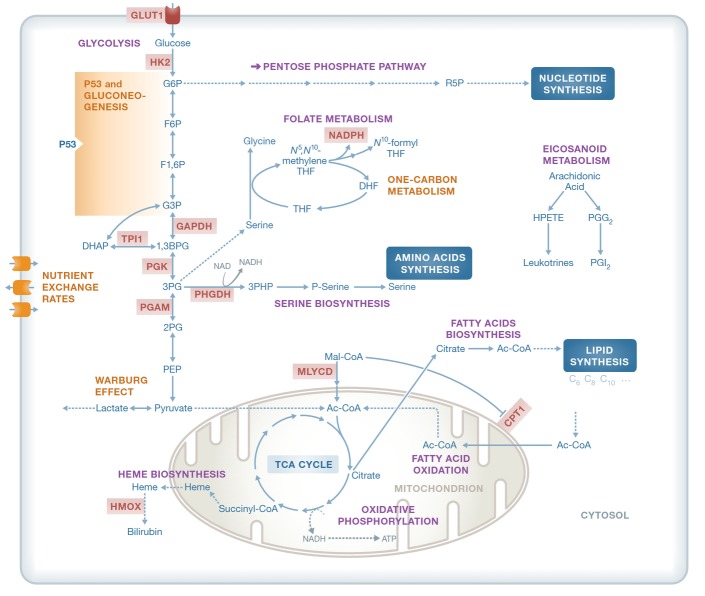
Metabolic processes, enzymes and metabolites that have been studied via Genome-scale metabolic modeling (GSMM) Some of the processes studied include the Warburg effect, the regulation of p53 on gluconeogenesis, one-carbon metabolism and nutrient exchange rates in cancer cell lines. A subset of the metabolic enzymes predicted by GSMM and validated experimentally appear in red. Additionally, the role of one-carbon metabolism in contributing to the cell's NADPH pool has been studied deeply. Leukotrienes and prostaglandins have been suggested as reporter metabolites in different cancer cell lines.

In recent years, many systems biology studies have been collecting molecular omics and phenotypic data for studying cancer. The availability of such high-throughput omics data provides the opportunity of integrating this data within a generic human GSMM to infer the metabolic activity state characterized by these measurements, in a *cell-specific* and *condition-dependent* manner (Jerby & Ruppin, 2012; Lewis & Abdel-Haleem, 2013) and, importantly, without the need to define a cellular objective function see ((Machado & Herrgård, 2014), Box 1). Omics integration has been mainly used toward two main goals: (i) characterizing the metabolic state of different cancer cells and studying fundamental cancer-related phenomena and (ii) identifying cancer metabolic drug targets and biomarkers in a context- and type-specific manner (Fig3).

### Studying cancer-related metabolic phenotypes

To describe the metabolic alterations in cancer, several GSMM studies have looked into alterations in central metabolism that are common among tumors, such as aerobic glycolysis (the Warburg effect) and enhanced biomass production and proliferation (Resendis-Antonio *et al*, 2010; Folger *et al*, 2011; Shlomi *et al*, 2011; Vazquez & Oltvai, 2011). By utilizing a metabolic model of central metabolism Vazquez *et al* (2010) and Vazquez and Oltvai (2011) have shown that at low glucose uptake rates mitochondrial respiration is indeed the most efficient pathway for ATP generation. However, above a threshold of metabolic rate, activation of aerobic glycolysis is favoured because it provides higher ATP production per volume density than mitochondrial oxidative phosphorylation. Studying this phenomenon on a genome scale Shlomi *et al* (2011) have shown that the Warburg effect may be a direct consequence of the metabolic adaptation of cancer cells to increased biomass production rate. Their model captured a three-phase metabolic behavior that is observed experimentally during oncogenic progression. Recently, Yizhak *et al* have studied the role of the Warburg effect in supporting cancer cell migration, trying to extend our understanding of this phenomenon beyond its association with cellular proliferation. Computing the predicted ratio of glycolytic ATP flux rate versus the oxidative one across different cancer cell lines, a strong positive significant association with cell migration was identified, thereby suggesting an additional role of the Warburg effect in supporting later stages of tumor progression. Apart from the Warburg effect, the generic human model has been recently used to study the relative contribution of different metabolic pathways to NADPH production, showing that 40% of NADPH production is predicted to come from one-carbon metabolism mediated by tetrahydrofolate (THF), an observation that was thoroughly experimentally verified in this study (Fan *et al*, 2014; Fig3).

Other GSMM studies have integrated cancer omics data to characterize a cancer-specific metabolic behavior. The first step in this direction was taken by Folger *et al*, who have generated a generic genome-scale model of cancer metabolism based on a core set of cancer-related enzymes. This model captured the main metabolic functions shared by many cancer types and has shown to successfully identify genes that are essential for tumor growth (Folger *et al*, 2011). Moving toward tumor-specific GSMMs (Agren *et al*, 2012; Wang *et al*, 2012b), Agren *et al* have constructed metabolic models for 16 cancer types and their parent tissues, predicting metabolites that are significantly more involved in the metabolism of cancer cells, such as polyamines, isoprenoid and eicosanoid metabolites, in correspondence with recent reports in the literature (Fig3). Later on, the same group has used a more comprehensive human model reconstruction (Mardinoglu *et al*, 2013a) to build tumor-specific models for breast, bladder, liver, lung and renal cancer tissues based on their proteomic signatures. A topological network analysis of these models has shown that clear cell renal cancer demonstrates a metabolic shift that associates differential down-regulation of one-carbon metabolism enzymes with poor clinical outcome. Interestingly, specific defects in nucleotides, one-carbon and glycerophospholipid metabolism that are unique to this type of cancer could be explained by loss of heterozygosity in multiple metabolic genes adjacent to the von Hippel-Lindau (VHL) tumor suppressor, which is frequently deleted in this type of cancer (Gatto *et al*, 2014). An alternative custom-built set of 26 tumor models was used by Wang *et al* (2012b) to identify tumor-enriched pathways according to model-based flux distributions, going beyond those predicted using differential gene expression alone. Lastly, focusing on specific cancerous mutations Goldstein *et al* (2013) have used the generic human metabolic network to characterize the metabolic state of liver-derived cancerous cells with a varying p53 status, with their results suggesting that P53 diverts glucose away from growth-promoting pathways to gluconeogenesis, thereby inhibiting oncogenesis (Fig3).

Moving toward the analysis of larger cohorts of cancer cells, Dolfi *et al* (2013) have integrated cell volume measurements, estimated DNA content and exchange fluxes of the NCI-60 cell lines, and showed that nutrient exchange rates are correlated with cell proliferation only when the variability in cell size is taken under consideration (Fig3). At the intersection of cancer cell lines and clinical samples, Feizi *et al* have identified metabolic subnetworks based on the generic human model and gene expression levels collected from both the NCI-60 cell lines collection and colon cancers. Interestingly, many of the major subnetworks that were found to be positively and significantly associated with cancer cell line proliferation were found to be negatively associated with patients' survival (Feizi & Bordel, 2013). On the clinical side, Jerby *et al* have used gene expression and proteomics derived from breast cancer patients to perform a GSMM analysis of their tumors, showing that advanced breast cancers have an increased flux in glycolysis, lactate production and ROS detoxification. The model's predictions of proliferation rates, ROS production and biomarkers were experimentally validated. The latter investigation also revealed a fundamental inherent stoichiometric trade-off between serine and glutamine metabolism, which underlies key metabolic differences between the ER+ and ER− subtypes (Jerby *et al*, 2012).

### Identifying perturbations targeting cancer metabolism

The analysis of different cancerous cells and states provides the opportunity for predicting new cytotoxic drug targets through the genome-scale predicted effects of various cellular perturbations. A deeper analysis involving richer datasets can extend upon that and address more complex challenges such as drug selectivity and drug resistance, as well as the targeting of other metabolically related cancerous alterations.

Several studies aiming to accomplish these goals have been published in recent years. The generic cancer model built by Folger *et al* (2011) has been used to predict 52 cytostatic drug targets, of which 40% were targeted by either approved or experimental anticancer drugs at the time of its publication. The same approach has later been used by Frezza *et al* (2011b) to build a cancer cell-specific model of newly characterized genetically modified kidney mouse cells in which *Fh1* has been deleted, thus studying the germline mutation of fumarate hydratase *(FH)* responsible for HLRCC. The HLRCC model has been used for identifying selective drug targets through a synthetic lethality (SL) approach and led to the identification of enzymes along the heme biosynthesis pathway as potential SL-pair targets of *FH*. Indeed, experimental validation of such a target, *HMOX,* was shown to selectively kill FH-deficient cells while sparing the normal ones (Fig3).

Exploring the effects of currently available drugs Facchetti *et al* (2012) have developed a novel GSMM-based method to investigate potential synergies between metabolic drugs, thus predicting optimal combinations of anti-cancer drugs with minimal side effects on normal human cell. Li *et al* (2010) have similarly utilized information on existing drugs and investigated flux predictions for the NCI-60 set of cell lines. This investigation identified drug-reaction interactions that were then used to predict new targets for approved anti-cancer drugs. Further along these lines Asgari *et al* (2013) have performed a topological analysis for 15 normal and cancer-specific metabolic networks, showing that approved anticancer metabolic drugs are not associated with highly connected enzymes, as may have been expected.

Recently, Agren *et al* have searched for antimetabolites aiming to target multiple enzymes simultaneously. Applying this approach for personalized models of six hepatocellular carcinoma patients has predicted 147 such potential antimetabolites. Out of which, the analogs of l-carnitine were studied experimentally by examining the effect of perhexiline, an inhibitor of carnitine palmitoyltransferase 1 (CPT1) on the proliferation of a HepG2 cell line, showing reduced viability of these cells (Agren *et al*, 2014) (Fig3). In a recent study, cell-specific models of a few hundreds of normally proliferating and cancerous cell lines were built by the quantitative integration of their gene expression levels (Yizhak *et al*, 2014a). These cell-specific models were then shown to successfully predict metabolic phenotypes on an individual level, including cellular proliferation rate, biomarkers and drug response. These models were also used to identify selective drug targets, which has led to the experimental validation of a top predicted selective target, *MLYCD*, in both a leukemia and kidney cancer cell lines versus their normal counterpart. A mechanistic investigation of the cytotoxic effect induced by *MLYCD* deficiency has demonstrated the potential role of oxidative stress in this process (Yizhak *et al*, 2014a; Fig3). As briefly described above, these cancer models were then used to predict the ratio between glycolytic and oxidative ATP production rate, showing its positive association with cell migration. Following, a dozen of novel gene perturbations that were predicted to reduce this ratio were found experimentally to significantly attenuate cell migration, while having almost no effect on cellular proliferation, as predicted. Importantly, such targets may reduce cytotoxic-related clonal selection of more aggressive cancer cells and the likelihood of emerging resistance (Yizhak *et al*, 2014b; Fig3).

Taken together, these studies clearly demonstrate the considerable potential value of GSMMs in deciphering the metabolic underpinnings of different tumorigenic phenotypes. Those include the fundamental characteristic of increased cell proliferation, as well as less metabolically direct cancerous phenotypes such as increased cellular migration and invasion. In addition, the various drug targets and biomarkers already revealed by GSMM-based studies and further validated experimentally testify for their ability to capture network-wide level effects that could not have been identified by data analysis alone.

## Future challenges in the modeling cancer metabolism—what lies ahead?

While there has been a remarkable progress in the last 4 years in the genome-scale modeling of cancer metabolism, additional challenges lie ahead in terms of both methodological and translational advancements. These include the utilization of richer datasets from both cell lines and clinical samples, the consideration of different cellular regulatory mechanisms, the modeling of cancer cell environment including its interactions with surrounding cells, and studying and assessing the potential of emergent drug resistance to metabolic cancer drugs. Further in the future, as more detailed kinetic information on specific central metabolism in humans is gathered, one may begin to address the challenge of building integrated kinetic and stoichiometric models of cancer metabolism.

### Integrating additional omics data sources

As reviewed above, the GSMM framework is a platform for omics data integration that can be of significant value. Nonetheless, transcriptomics and proteomics have been the main data source for deciphering metabolic phenotypes, while other data sources have been rarely used.

**Figure 4:**
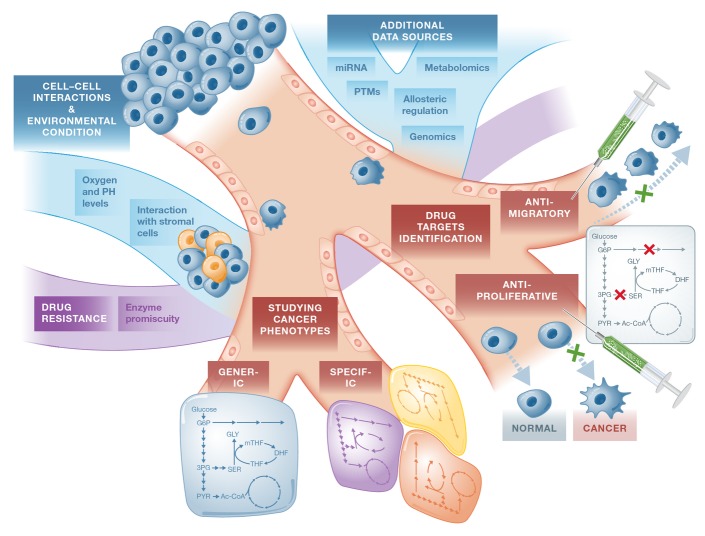
Current and future applications of GSMMs In the context of cancer metabolism, Genome-scale metabolic modelings (GSMMs) have been applied for studying fundamental cancer phenotypes that are either generic or tumor/cell-specific and for identifying drug targets that inhibit cancer-related phenotypes such as proliferation and migration in a specific and selective manner. GSMMs can also be used for addressing emerging challenges in cancer therapy such as drug resistance. Furthermore, the analysis of GSMMs can be extended by integrating additional omics data such as genomics and metabolomics and by utilizing the information on post-transcriptional and post-translational integration as well as incorporating allosteric regulation effects. Another challenge is the modeling of the interaction between cancer cells and supporting cells in their environment. Environmental effects can also be modeled by integrating structural analysis and predicting the effects of environmental conditions (which cannot be modeled directly) on enzyme activities.

New technology for next-generation sequencing (NGS) has enabled a systematic cataloging of cancer genomes through national and international genomics projects (Simon & Roychowdhury, 2013). The Cancer Genome Atlas and the International Cancer Genomics Consortium are examples for such comprehensive resources where mutational signatures and potentially new therapeutic targets across cancer types have been identified (Alexandrov *et al*, 2013; Yang *et al*, 2013a). By focusing on the subset of mutated metabolic enzymes and evaluating their effect on protein function, one can potentially use these datasets to model multiple cancer subtypes and identify their unique metabolic vulnerabilities (Fig4). A first step in this direction has been recently taken by Nam *et al* (2014). In this study, the authors integrated genetic mutation data from more than 1,700 cancer genomes along with their gene expression levels. Predicted flux changes between normal and cancer cells were then evaluated by simulating loss-of-function mutations in metabolic enzymes, leading to the prediction of 15 predicted oncometabolites, reassuringly including the well-known oncometabolites succinate and fumarate.

Apart from genomics, metabolomics is an additional accumulating data resource for studying cancer biology. Metabolomic profiles of cancer cells have been widely used for the past several years to distinguish between different cell lines and tumor types both *in vitro* and *in vivo* (Florian *et al*, 1995; Tate *et al*, 1998). Furthermore, cancer-associated mutations in certain metabolic genes were found to induce an abnormal accumulation of oncometabolites (Yang *et al*, 2013b). For instance, as already described above, mutations in IDH1 and IDH2 result in the generation of 2-hydroxyglutarate (2HG), which alters gene transcription through DNA modifications and histone methylation (McCarthy, 2013). The ability to both integrate and predict metabolite concentrations on a genome-scale level is therefore of major importance in studying cancer metabolism (Fig3). While information on extracellular metabolites has been used to constrain a given GSMM (Agren *et al*, 2012; Schmidt *et al*, 2013), the prediction and/or integration of intracellular metabolite levels requires the usage of thermodynamic information and the knowledge of the kinetic parameters (Yizhak *et al*, 2010; Cotten & Reed, 2013), which are largely unknown. The utilization of metabolomic data for analyzing GSMMs therefore calls for new, more sophisticated methodologies designed to address these emerging challenges.

### Accounting for different cellular regulatory mechanisms

The great majority of GSMM-based cancer studies rely solely on the metabolic–stoichiometric aspects of the human network and its integration with different omics datasets. The next step extending upon that is the integration of different regulatory mechanisms, including transcriptional and post-transcriptional regulation (Fig4). Methods for developing integrated metabolic–regulatory GSMMs have already been developed and studied in microorganism (Covert *et al*, 2004; Herrgard *et al*, 2006; Shlomi *et al*, 2007). The computational machinery for achieving this goal can therefore be readily used for higher organisms as well. Nonetheless, information on the architecture of the human regulatory network and its complexity has only recently been starting to accumulate through projects such as the ENCODE (Consortium, 2012). Utilizing these newly incoming rich data resources to reconstruct a human metabolic–regulatory network model is of tremendous potential in accelerating the modeling of human metabolism in general, and cancer metabolism in particular.

Additional genomic regulatory information that can be used to account for different cancerous cellular states is microRNA (miRNA) levels and epigenetic modifications. miRNA alterations were already found to be involved in the initiation and progression of human cancer, as reflected by the widespread differential expression of miRNA genes in malignant compared to normal cells (Calin & Croce, 2006). Recently, Wu & Chan (2014) have integrated miRNA-target prediction, metabolic modeling and context-specific gene expression data to predict therapeutic miRNAs that could reduce the growth of cancer. This approach has been applied to human hepatocellular carcinoma (HCC) wherein overexpression of each miRNA was simulated to predict their ability to reduce cancer cell growth. Remarkably, the overall accuracy in predicting the miRNAs that could suppress metastasis and progression of liver cancer was > 80%. An additional type of regulation that has not been widely studied yet is that of allosteric regulation. The incorporation of allosteric (in)activation information concerning metabolic enzymes is currently missing from the basic GSMM analysis and can certainly boost its predictive power (Fig3).

### Modeling cancer cells environment and interactions

While many studies have focused on growing cancer cells *in vitro* and out of their tumorigenic context, it is now widely accepted that the tumor microenvironment plays an important role in defining and reprogramming cancer cell metabolism (Morandi & Chiarugi, 2014). The computational study of cell and tissue interactions via GSMMs has already been demonstrated in both microorganisms and human tissues (Bordbar *et al*, 2011; Freilich *et al*, 2011; Zomorrodi & Maranas, 2012), but has not been explored in the context of cancer cells and supporting cells in their environment. Modeling the dynamic exchange of material between these different cells can bring us closer to a more accurate modeling of tumors *in vivo* and reveal metabolically related phenotypes that could not have been discovered by the modeling of each cancer cell alone (Fig4).

Apart from the interaction with other cells in their microenvironment, cancer cells are also exposed to varying oxygen and pH levels. These factors play a key role in tumor development and are known to affect tumor cell metabolism (Helmlinger *et al*, 1997). While oxygen and nutrient availability in general can be simulated directly via GSMMs, the simulation of environmental factors such as pH is less straight forward. One possible approach for addressing this challenge is by applying structural analysis to predict the effect induced by pH levels over the activity of metabolic enzymes (Fig4). Interestingly, a conceptually somewhat analogous analysis has been applied to study *Escherichia coli* response to diverse temperatures, revealing protein activities that limit network function at higher temperatures and providing mechanistic interpretations of mutations found in strains adapted to heat (Chang *et al*, 2013).

### Studying the emergence of resistance to metabolic drug targets

Resistance to chemotherapy and molecularly targeted therapies is a major problem facing current cancer research, and the mechanisms for its acquirement are diverse (Gottesman, 2002). GSMMs can be utilized in this context to identify promiscuous functions of existing metabolic enzymes, thus revealing alternative pathways capable of bypassing the targeted oncogenic reaction(s). Furthermore, this approach can be used to identify gain-of-function enzyme mutations and increase our understanding of enzymes' catalytic side activities (Fig4). Promiscuous functions of metabolic enzymes have already been studied by GSMM of *Escherichia coli,* both revealing fundamental features of these enzymes (Nam *et al*, 2012) and identifying novel metabolic pathways that produce precursors for cell growth under diverse environmental conditions (Notebaart *et al*, 2014). In addition to that, the GSMM framework also enables the simulation of multiple perturbations simultaneously and can thus facilitate the investigation of drug combinations therapy and SL-based treatments. These investigations provide an opportunity for achieving greater selectivity and specificity, offering tremendous potential for improved prognoses.

In closing, one should note that in addition to GSMMs, other more early approaches exist for the modeling of biological processes, including large-scale topological and Boolean networks, and the more classic, small-scale analyses through ordinary differential equations (ODE) (Resendis-Antonio *et al*, 2014). The detailed review of these approaches is beyond the scope of the current paper. Here we just note in brief that topological networks have been used for studying how genes coordinate their expression in various biological states, and were applied to identify drug targets in different contexts, including glioblastoma, breast and cervical cancers (Horvath *et al*, 2006; Higareda-Almaraz *et al*, 2011; Wu & Stein, 2012). On the other hand, Boolean network analyses involve the modeling of the dynamics of transcription regulatory and signaling networks (Wang *et al*, 2012a), and were used for identifying genes driving the transitions between different tumor progression stages, and determining driver mutations that promote cancerous phenotypic transitions as a function of the cell's microenvironment (Fumiã & Martins, 2013; Srihari *et al*, 2014). ODE models were mainly used in this context for studying the dynamics of tumor growth (Laird, 1964) and understanding tumors' response to therapy (Lankelma *et al*, 2013). Though challenging, the combination of these different approaches can bring us closer toward the holy grail of whole-cell modeling, which we proceed to discuss in our concluding remarks.

## Conclusions

As evident, genome-scale metabolic modeling provides valuable insights into cancer metabolism and holds promise for many more interesting and clinically relevant applications to come. Importantly, GSMM is a stepping stone for whole-cell modeling, and this vision, which was already firstly realized by Karr *et al* (2012) in bacteria, should inspire us to aim at modeling the entire cellular dynamics of different cancer cells. While clearly cancer cells represent a much more complex system, we should bear in mind that the enormous amount of data accumulated by the scientific community about cancer, and the pace in which it grows, is orders of magnitude larger than any other cellular system. The initial strides discussed here for GSMMs demonstrate that, perhaps, “yes, we can.”
